# Binding of Lysozyme to Spherical Poly(styrenesulfonate) Gels

**DOI:** 10.3390/gels4010009

**Published:** 2018-01-16

**Authors:** Martin Andersson, Per Hansson

**Affiliations:** Department of Pharmacy, Uppsala University, Box 580, SE-75123 Uppsala, Sweden; martin.andersson@ge.com

**Keywords:** polyelectrolyte gel, protein, microgel, microscopy, X-ray scattering, mechanism, kinetics

## Abstract

Polyelectrolyte gels are useful as carriers of proteins and other biomacromolecules in, e.g., drug delivery. The rational design of such systems requires knowledge about how the binding and release are affected by electrostatic and hydrophobic interactions between the components. To this end we have investigated the uptake of lysozyme by weakly crosslinked spherical poly(styrenesulfonate) (PSS) microgels and macrogels by means of micromanipulator assisted light microscopy and small angle X-ray scattering (SAXS) in an aqueous environment. The results show that the binding process is an order of magnitude slower than for cytochrome c and for lysozyme binding to sodium polyacrylate gels under the same conditions. This is attributed to the formation of very dense protein-rich shells in the outer layers of the microgels with low permeability to the protein. The shells in macrogels contain 60 wt % water and nearly charge stoichiometric amounts of lysozyme and PSS in the form of dense complexes of radius 8 nm comprising 30–60 lysozyme molecules. With support from kinetic modelling results we propose that the rate of protein binding and the relaxation rate of the microgel are controlled by the protein mass transport through the shell, which is strongly affected by hydrophobic and electrostatic interactions. The mechanism explains, in turn, an observed dependence of the diffusion rate on the apparent degree of crosslinking of the networks.

## 1. Introduction

Complex formation between proteins and polyelectrolytes of opposite charge in aqueous solution has been a topic of scientific interest since the middle of the last century, arising from the need to develop methods for protein separation [1]. The topic has since then been attracting a growing interest [2,3,4,5,6,7]. The interaction between the components is typically dominated by electrostatic forces and influenced by variations of pH and ionic strength [8]. Other important factors are polyion chain stiffness [9] and linear charge density [10], the net charge and distribution of charges [11] and hydrophobic patches on the protein surface [2], factors that may influence both the polyion–protein and protein–protein interactions.

The present paper deals with the interaction between responsive polyelectrolyte networks of microscopic/mesoscopic size (50–100 µm) and proteins of opposite charge. Our interest in such systems derives both from their tentative use as drug delivery systems [12,13] and from intriguing biological phenomena, such as protein-assembly/sorting mediated by polyelectrolyte networks in protein secreting cells [14,15].

The swelling of polyelectrolyte networks depends to a large extent on the osmotic pressure exerted by the counterions. The exchange of monovalent counterions for multivalent proteins is therefore expected to be followed by a gel volume decrease. This was demonstrated quite early for the case of small cationic proteins binding with polyacrylate (PA) networks of macroscopic size (~1 mL) [16,17,18,19,20]. It was also shown that the gels may display a core/shell structure during protein uptake, with dense protein–polyelectrolyte complexes making up the shell. Similar observations have been made in other systems where highly responsive microgels or macrogels interact with proteins [21,22,23,24], synthetic polyions [25,26], polypeptides [27,28,29], as well as micelle-forming surfactants [30,31,32,33,34].

Lysozyme and cytochrome c (cyt c) are small basic globular proteins of similar size and net charge. Despite their similarity they display quite different interaction patterns with PA macrogels [16] and microgels [22,24]. While cyt c diffuses fast inside PA microgels to end up uniformly distributed at long times [22], lysozyme has been shown to form a rigid and long-lived shell arresting the gel in a semi-swollen state, believed to be caused by the aggregation of lysozyme inside the network [23,24]. It is known from the literature that lysozyme is prone to self-aggregate in screening aqueous environments [35,36,37], but that cyt c largely lacks this property [38]. This suggests that the difference between them in the gel systems can be related to protein–protein interactions rather than protein–polyion interactions [22]. Information of this kind is important for applications of microgels in, e.g., drug delivery and encapsulation technologies. In the present paper we investigate this further by studying the binding of lysozyme to sodium poly(styrenesulfonate) (PSS) microgel spheres by means of micropipette-assisted microscopy. PSS is known to interact electrostatically and hydrophobically with lysozyme [39,40,41] and with cationic surfactant micelles [42,43,44,45], in contrast to PA which is expected to display mainly electrostatic interactions. One motive behind the study is thus to investigate how the hydrophobic backbone of PSS affects the binding and aggregation of lysozyme in gels. Another is to investigate the role of the crosslinks in the network. The study includes also a small-angle X-ray scattering (SAXS) investigation of the microstructure of the complexes formed in PSS macrogels, and a minor study of cyt c binding to PSS macro- and microgels. It will be shown that the binding process is very slow compared to that observed in related systems which can be attributed to the formation of very dense protein-rich shells in the outer layers of the microgels with low permeability to the protein. The results will be discussed and interpreted in the light of recent results for linear PSS/lysozyme mixtures, which are particularly well characterized due to a fortuitous situation with respect to contrast matching in small-angle neutron scattering (SANS) experiments [10,46,47,48,49].

## 2. Results and Discussion

### 2.1. Swelling Characteristics of Protein-Free Gels

**Figure 1:**
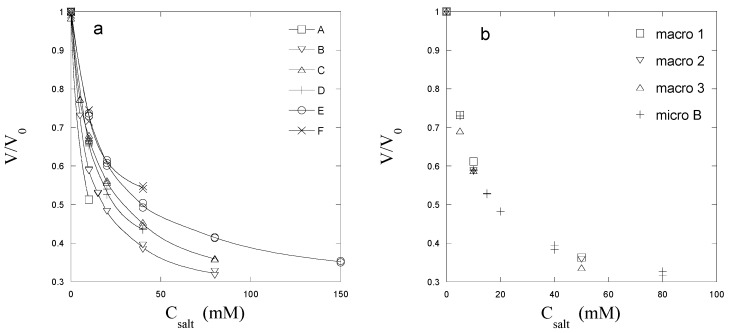
Gel volume (*V*) relative to that in pure water (*V*_0_) plotted vs. the concentration of NaCl in the solution (*C*_salt_). (**a**) Microgel A–F; (**b**) Microgel B (micro B) and three different macrogels from the same batch (macro 1–3).

Figure 1a shows the equilibrium volume of five individual PSS microgel species (A–F) plotted as a function of the concentration of NaCl in the solution (no protein present). The ordinate shows the gel volume (*V*) relative the volume in pure water (*V*_0_). Each particle responds to the variation of the salt concentration in a distinct way, related to the effective degree of cross-linking of the PSS network. The latter is expected to be determined by the average number of styrenesulfonate (SS) monomers between crosslinks and the presence of entanglements and ‘dangling’ chains. In this paper, we will characterise this by the ratio *V*_0_/*V*_10_, where *V*_10_ is the gel volume in 10 mM NaCl. Pictures of the microgels in NaCl solutions of different concentrations can be found in the Supplementary Material S1. It will be shown below that *V*_0_/*V*_10_ is an indicator of how a microgel behaves during uptake of lysozyme from solutions containing 10 mM NaCl, the latter arbitrarily chosen as the standard condition for the loading of microgels in the present study. Figure 1b shows the salt response of microgel B and three macrogel species. All gels, including the microgel, display a similar overall salt response and have nearly the same *V*_0_/*V*_10_-value. The fact that the response of the micro- and the macrogels are very similar in a wide range of salt concentrations suggests similar network structures.

**Figure 2:**
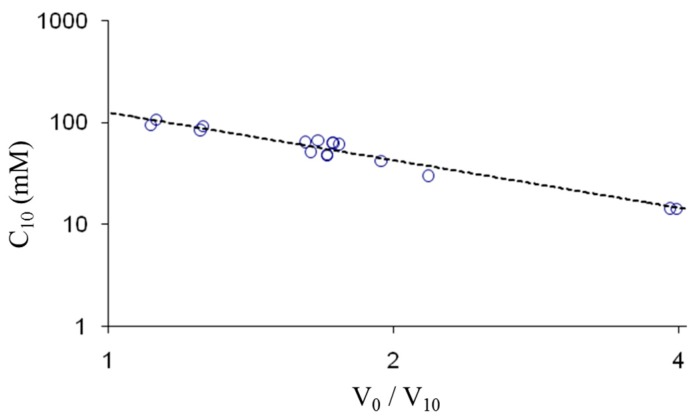
The concentration of network charges in PSS macrogels in 10 mM NaCl solution (*C*_10_) plotted vs. the ratio between the gel volume in pure water (*V*_0_) and in 10 mM NaCl (*V*_10_). The dashed line is a fit to the data, $C_{10}\left(\mathrm{mM}\right)=125{\left(V_{0}/V_{10}\right)}^{-1.552}$.

Another important characteristic is the concentration of network charges in the microgels. An estimate can be obtained from comparison with the swelling properties of macrogels. The result of a determination of the concentration of PSS in the macrogels is presented in Figure 2 as a log–log plot of *C*_10_ vs. *V*_0_/*V*_10_, where *C*_10_ is the concentration of network charges in gels equilibrated in 10 mM NaCl solution. The plot shows that the data for different macrogels with widely different *V*_0_/*V*_10_ values conforms to a linear scaling law. The similarity between micro- and macrogels displayed in Figure 1b suggests that the relationship should be valid also for the microgels. The *C*_10_-values calculated from the *V*_0_/*V*_10_ values for the microgels by means of the relationship shown in Figure 2 is given in Table 1 (estimated error: ±10%). Justification of this procedure is provided in the Supplementary Material S2, which shows that a theoretical model capable of capturing the macrogel scaling law in the relevant range in Figure 2 can be fitted to the salt response data for the microgels with realistic values of the model parameters Figures S7 and S8. The magnitude of *V*_0_/*V*_10_ also reflects the ease with which a microgel deforms elastically when impinged on by the tip of a thin glass rod using the micromanipulator. This characteristic was used as a guide to select microgels covering a wide range of responsiveness.

### 2.2. Lysozyme Binding to Microgels

**Figure 3:**
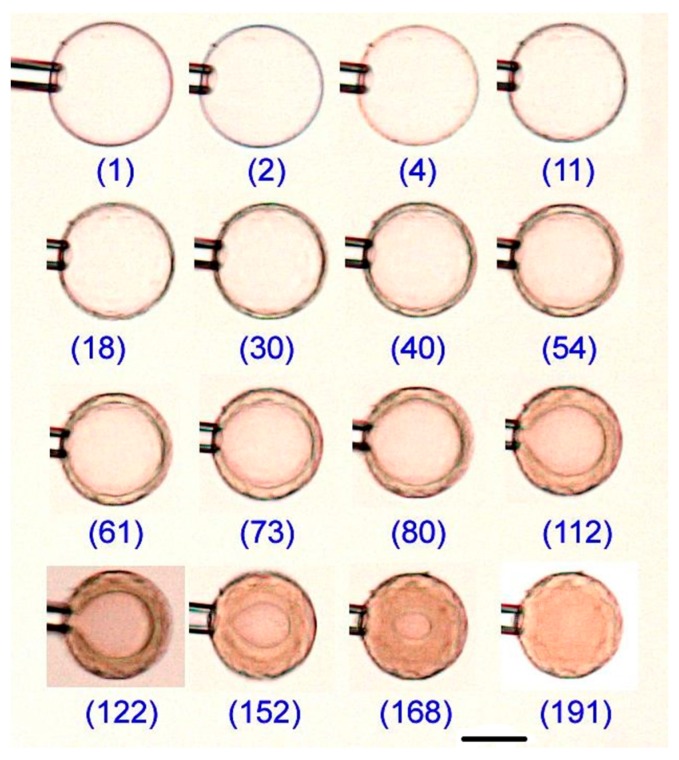
Microscopy images of microgel B at different times during deswelling in 800 mg/L lysozyme solutions (NaCl/tris buffer; pH 8.0, I = 10 mM). Numbers indicate the time in minutes after exposure to the protein solution. The tip of the micropipette is seen to the left of the microgels. Scale bar = 50 µm.

**Figure 4:**
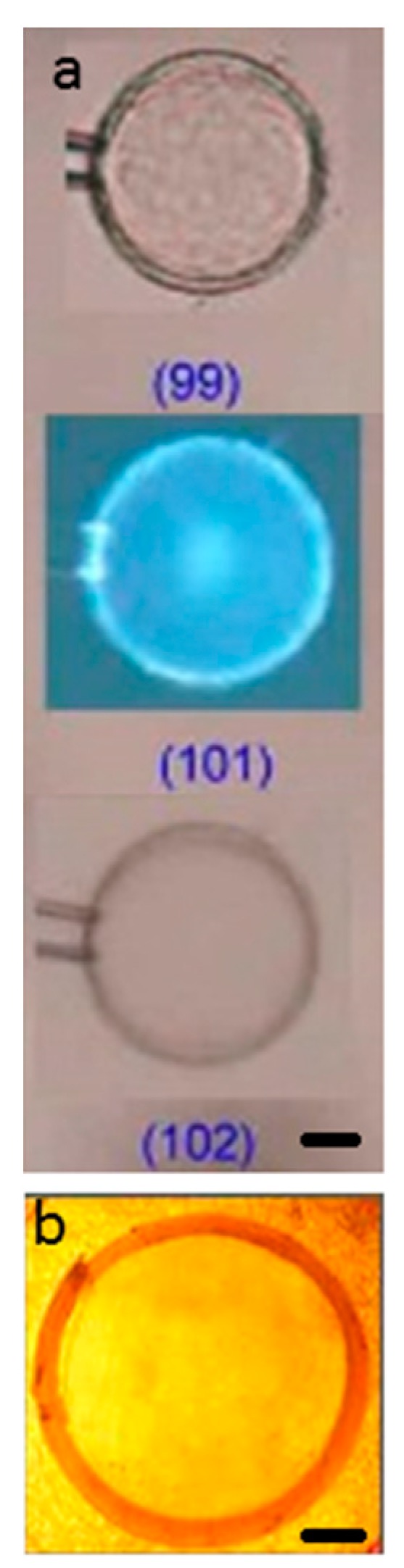
(**a**) Visualization of the protein rich shell in microgel E during deswelling in 500 mg/L lysozyme solutions (NaCl/tris buffer; pH 8.0, I = 10 mM). Shown are microscopy images taken with without condenser (top), with fluorescent light (middle), and with condenser (bottom) after 99, 102, and 101 min, respectively, after onset of experiment as indicated; scale bar = 25 µm. Numbers indicate the time in minutes after exposure to the protein solution; (**b**) photo of a thin slice through the PSS macrogel with lysozyme-rich shell (*β* = 0.55); scale bar = 2.5 mm.

The micromanipulator-assisted microscopy setup was used to investigate the volume response of the network and the evolution of internal structures in single PSS microgels exposed to lysozyme solutions. The fluid medium was NaCl/tris buffer (pH 8.0, I = 10 mM) in all experiments. Figure 3 shows pictures of microgel B during various stages of the binding process in 800 mg/L lysozyme. The other microgel systems showed the same qualitative behavior (a full set of images of all microgels are given in Figures S1–S5 in Supplementary Material S1). The two most striking features are (1) the formation of a surface layer (shell) in the gels and (2) the long time needed for the gels to relax in the new environment. A shell with a sharp boundary to the core is clearly visible after 20–30 min. However, already within a minute the contrast between the shells and the surroundings increased significantly indicating a densification of their structure. Figure 4a highlights the core/shell structure remaining after long time (~100 min) in microgel E, with the high packing density of the shell revealed by the intrinsic fluorescence of the protein.

**Figure 5:**
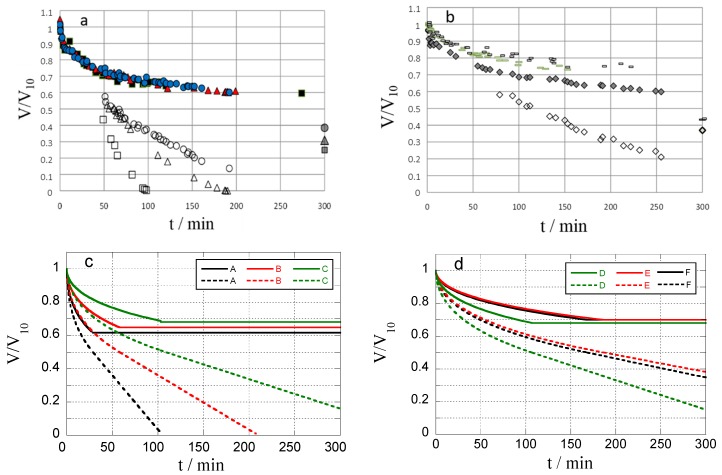
Relative volume (*V*/*V*_10_) of microgel and protein free gel core vs. time in lysozyme solution (NaCl/tris buffer; pH 8.0, I = 10 mM). (**a**,**b**): Experimental data for microgels A (squares), B (triangles) and C (circles) at *C*_lys_ = 800 mg/mL, and D (diamonds), E (long rods), F(short rods) at *C*_lys_ = 500 mg/mL. Predicted final volumes are arbitrarily positioned at 300 min in (**a**,**b**); (**c**,**d**): Theoretically calculated data for microgels A–F showing the gel volume (solid curve) and the core volume (dashed); curves are calculated by Equations (S12) and (S13) with input values of concentrations and microgel characteristics as in Table 1.

In all systems the core/shell boundary is seen to migrate inward, a process accompanied by the deswelling of the microgel. Figure 5a,b show how the relative volume (*V*/*V*_10_) varies with time in solutions containing 500 and 800 mg/L lysozyme, respectively. *V*_10_ is the microgel volume in 10 mM NaCl solution in the absence of protein. The time scale for the process is several hundred minutes, which can be compared with the typical time of 1–10 min for the binding of proteins [22,23,24], peptides [27,28,29], and surfactants [43,50,51,52,53] to microgels of similar size. In fact, the relaxation time is comparable to that of protein and surfactant binding to macrogels (~1 mL) [16,53]. The curves for gels A–D nearly overlap and relaxes to a quasi-plateau somewhat lower than the curves for gels E and F. The differences between the microgels appear more clearly when comparing the evolution of the core volume, also shown in Figure 5. (Note that core data at small times is missing since the resolution of the microscope only allowed accurate measurement of shell thicknesses after about 50 min.) Interestingly, the rate of core shrinking follows the order A > B > C > D, which correlates with the responsiveness of the microgels as measured by the ratio *V*_0_/*V*_10_ (Table 1). However, the vanishing of the core does not mean that the gels become homogeneous. This is evident from the picture taken after 191 min for microgel B in Figure 3, and further proved by fluorescence microscopy (Figures S1 and S4 in Supplementary Material S1). The behavior is reminiscent of the binding of lysozyme to polyacrylate (PA) microgels where an initial shell formation process was shown to be followed by core diffusion [24]. During the latter process, protein transported through the shell was proposed to aggregate in the core at a lower density than in the shell, but in such a way that a sharp boundary was maintained to the protein-free part of the core. A similar mechanism will be discussed below for the present gels. However, the time scale for both processes is much longer for PSS than for PA microgels; in the latter the total relaxation time is less than 5 min, even for lysozyme concentrations in the solution lower than the present ones.

Inhomogeneous shells remaining at long times after consumption of the protein-free core are also observed for microgels B and C in the present study. Careful inspection of the microscopy images suggests that the lysozyme-filled microgels can be arrange in the following order based on inhomogeneity: A > B > C > D. Support for this will be provided in a later section from theoretical calculations by means of a mass transport model.

Before proceeding we mention that substantial amounts of lysozyme (incorporated at 10 mM NaCl) remained in the microgels even after flushing in the flow tube with solutions of high ionic strength. For example, after a long time in contact with a 0.2 M NaCl solution, the volume of a lysozyme-loaded gel was found to be smaller than for the protein-free microgel in the same medium. In comparison, Li et al. found that all lysozyme pre-loaded into anionic oxidized starch polymer microgels was released under the same conditions [54]. In contrast, the dissolution of the PSS–lysozyme complexes started almost immediately when a 20 mM solution of the anionic surfactant SDS in 10 mM NaCl was added, and was followed by rapid and apparently complete unbinding of the protein, as can be seen in Figure S3 in Supplementary Material S1. The interaction between lysozyme and SDS is well documented in the literature [55]. It is known that a limited number of surfactant molecules can bind non-cooperatively to hydrophobic sites on the protein, thereby reducing its net positive charge. This should weaken the electrostatic interaction with the PSS chains. However, the inefficiency of salt to remove the protein from the gel indicates that the complexes are stabilized also by non-electrostatic forces. Thus, a preferential binding of the surfactant tail to hydrophobic sites on the protein may compete with and eventually rule out hydrophobic interactions between the protein and PSS (or the interaction between protein molecules).

### 2.3. Lysozyme Binding to PSS Macrogels

#### 2.3.1. Swelling Isotherm

**Figure 6:**
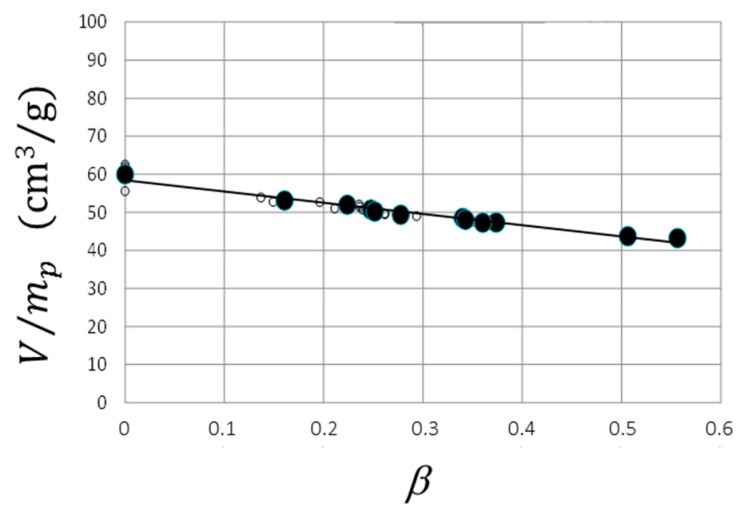
$V/m_{p}$ vs. *β* for PSS macrogels in lysozyme solution, pH = 8.0, I = 10 mM. The line is a linear fit to the data, $V/m_{p}\;=-31.6\beta +59$ (cm^3^/g).

The volume decrease in response to protein uptake was studied for spherical macrogels (~1 mL). The gels were placed in solutions containing limited amounts of lysozyme, which allowed the amount taken up by the gels to be calculated from measurements of the concentration remaining in the liquid. The liquid medium was the same as in the microgel study (I = 10 mM, pH = 8). During the experiment the lysozyme concentration in the solution decreased from 1000 to ca. 500 mg/L. The result is presented in Figure 6 as a plot of the gel volume per mass of dry NaPSS-network (cm^3^/g) vs. the protein-to-network charge ratio in the gel *β*, calculated assuming a +7.6 net charge of lysozyme [36]. The highest *β* shown (0.55) is the value reached when the experiment was interrupted after seven months. Gels examined at this stage were found to consist of a dense shell and swollen core. A picture of a 1 mm thick centerpiece slice taken from one of the gels (marked *β* = 0.55) is shown Figure 4b for comparison.

#### 2.3.2. Shell Composition

The following analysis shows that the composition of the shell can be obtained from measurements of the gel radius $r_{2}$ and the core radius $r_{1}$ in combination with the linear relationship in Figure 6.

Let $V_{s}$ and $V_{c}$ denote the volume of the shell and core, respectively, and *n_p_*_,*s*_ and *n_p_*_,*c*_ the number of moles of polyion charges in the shell and the core, respectively. Then we can write

(1) $$\frac{V}{n_{p}}=\frac{V_{c}+V_{s}}{n_{p}}=\frac{v_{c}n_{p,c}}{n_{p}}+\frac{v_{s}n_{p,s}}{n_{p}}=v_{c}\left(1-\frac{n_{p,s}}{n_{p}}\;\right)+v_{s}\frac{n_{p,s}}{n_{p}}=v_{c}+\left(v_{s}-v_{c}\right)\frac{n_{p,s}}{n_{p}},$$

where $n_{p}$ is the total number of moles of polyion charges, and $v_{s}=V_{s}/n_{p,s}$, $v_{c}=V_{c}/n_{p,c}$. The overall protein-to-network charge ratio is defined as

(2) $$\beta =\frac{Zn_{lys}}{n_{p}}$$

where *Z* is the net charge of the protein and $n_{lys}$ is the number of moles of protein molecules in the gel. Let $f_{s}$ be the polyion-to-protein charge ratio in the shell

(3) $$f_{s}=\frac{n_{p,s}}{Zn_{lys,s}},$$

where *n_lys,s_* is the number of moles of protein molecules in the shell. Then one obtains from a combination of Equations (1)–(3):

(4) $$\frac{V}{n_{p}}=v_{c}+\left(v_{s}-v_{c}\right)\frac{n_{lys,s}}{n_{lys}}f_{s}\beta .$$

When the core is free from protein ($n_{lys,s}$ = $n_{lys}$) and $v_{c}$ is independent of β, Equation (4) becomes:

(5) $$\frac{V}{n_{p}}=v_{0}+\left(v_{s}-v_{0}\right)f_{s}\beta ,$$

where $v_{0}$ is the initial gel volume ($V_{0}$) divided by $n_{p}$. Under such conditions we also have:

(6) $$\frac{v_{s}}{v_{0}}=\frac{V_{S}}{V_{0}-V_{c}}=\frac{r_{2}^{3}-r_{1}^{3}}{R_{0}^{3}-r_{1}^{3}},$$

where $R_{0}$ is the initial gel radius. As an alternative to (5) one can write:

(7) $$\frac{V}{m_{p}}=\frac{v_{0}}{M_{p}}+\frac{v_{s}-v_{0}}{M_{p}}f_{s}\beta ,$$

where $m_{p}$ is the dry mass of the polyelectrolyte network and $M_{p}$ is the molar mass of a charged unit of the network. The linear relationship between the specific gel volume and β in Figure 6 gives credit to the assumptions behind Equations (6) and (7). A linear least-square fit of Equation (7) to the experimental data is included in Figure 6. The composition of the shell is described by $f_{s}$ and $v_{s}$, which can be determined from Equations (6) and (7) by using the parameters obtained from the fit together with the estimates of $r_{1}/R_{0}$ and $r_{2}/R_{0}$ for the gel at *β* = 0.55. The result is $f_{s}$ = 0.97 and $v_{s}$ = 0.0054 m^3^/mol, from which one obtains that the concentration of polyelectrolyte charges and lysozyme net charges in the shell is 184 and 191 mM, respectively, and that the shell contains 60 wt % water. By describing the lysozyme molecule as an ellipsoid [56] with dimensions 3.0 × 3.0 × 4.5 nm^3^ the protein volume fraction becomes 0.32. We have also calculated the expected equilibrium microgel volumes (*V*_*β*=1_/*V*_10_) based on the assumption that the microgels become homogeneous with the same composition as the shell in the macrogel. The result is given in Table 1 and arbitrarily indicated at 300 min in Figure 5a,b.

#### 2.3.3. Shell Microstructure

**Figure 7:**
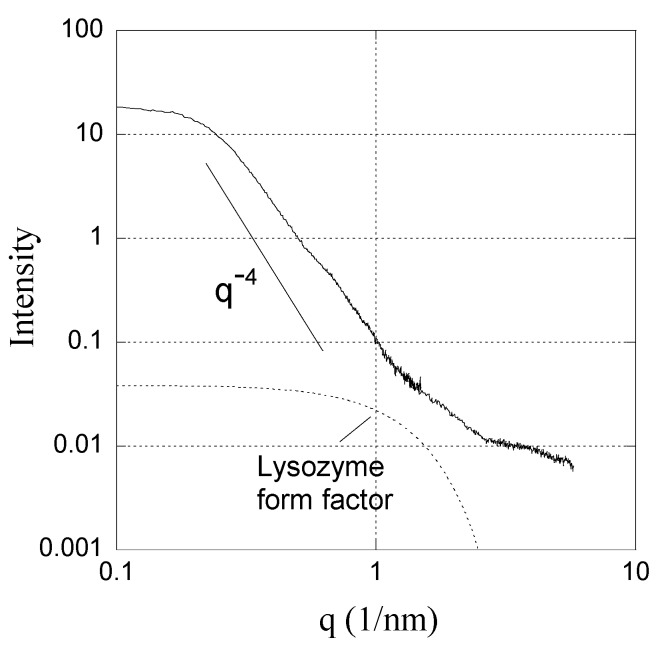
Synchrotron SAXS data for the shell formed by lysozyme in a PSS macrogel (*β* = 0.55). The form factor of lysozyme [57] is included for reference.

The shell of the macrogel (*β* = 0.55) was examined with synchrotron SAXS. The resulting scattering profile is shown in Figure 7. In the low q-range there is a plateau followed by a Porod law (*q*^−4^) region, indicative of large objects of finite size. A Guinier plot of the data is linear at low q (see Figure S9 in Supplementary Material S3). From the slope of the Guinier plot the radius of gyration is determined as 6.4 nm, which corresponds to a radius of 6.4 × (5/3)^½^ nm = 8.3 nm for a homogeneous sphere. Nearly the same value (8.0 nm) is obtained by fitting the form factor of a sphere to the scattering profile at low *q*. Interestingly, the size of the object is of the same order of magnitude as the “primary complex” described by Cousin, Gummel and co-workers, who studied mixtures of lysozyme and linear PSS by means of SANS [10,46,47,48,49]. In charge stoichiometric mixtures at low ionic strength, comparable to the one used here, they reported a radius of 7.3 nm. In contrast to what we observe, the complexes were found to be part of higher order aggregates with fractal geometry, giving rise to strong scattering at low *q* scaling as *q*^−2.1^ or *q*^−2.5^, but the composition of the complexes was remarkably independent of the overall composition of the mixture; the volume fraction of lysozyme was always about 0.3 and the polyion-to-protein charge ratio near one, similar to what is observed here. The high protein concentration resulted in a strong correlation peak at 2 nm^−1^ when contrast matching was utilized to display only the lysozyme scattering. The same peak, albeit weaker, could be seen also when the contrast was changed to show only the PSS scattering. As pointed out by the authors, the corresponding correlation distance (~3 nm) is equal to the closest center–center distance between lysozyme molecules. (Strong correlation between protein molecules in the complexes was always the case, except for mixtures with sufficiently long PSS chains at polyion-to-protein charge ratios >1, where overlapping PSS chains formed a mesh in which the protein was believed to act as cross-links between the chains).

Since the average volume fraction of lysozyme in the shell investigated by us is about the same as that in the primary complexes with linear PSS, the volume fraction in the complexes in the shell must be even higher. For example, by assuming random close packing of spherical complexes (volume fraction = 0.64) the internal volume fraction of lysozyme in the primary complexes would be 0.5, i.e., near the freezing value for hard spheres [58]. 

The lysozyme aggregation number *N* can be calculated as $N=\emptyset V_{compl}/V_{lys}$, where is ∅ the volume fraction of lysozyme in the complex, $V_{compl}$ is the volume of the complex, and $V_{lys}$ is the volume of a lysozyme molecule (21 nm^3^). By taking $V_{compl}$ as the volume of a sphere with radius 8 nm (see above) we obtain *N* ≈ 10^2^∅. Since ∅ must be larger than the average volume fraction in the shell and is not likely to exceed 0.6, we estimate that *N* is between 30 and 60.

**Figure 8:**
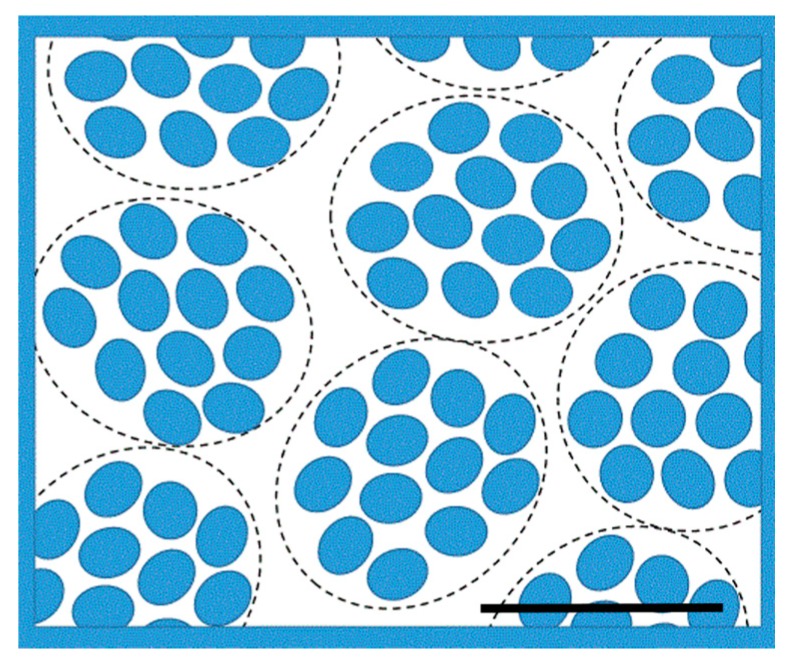
Schematic drawing of the shell microstructure revealed by SAXS showing individual lysozyme molecules (blue) arranged in dense lysozyme-PSS complexes of finite size. PSS network omitted for clarity. Bar = 16 nm.

The absence of correlation peaks in the SAXS spectrum is not necessarily in conflict with the high volume fraction since the contrast depends on variations of the electron density. In a structure of proteins packed closely together, with PSS chains filling the space between them, the electron density is expected to be fairly uniform, making the interior structure of the complexes invisible in a SAXS experiment. The absence of correlation peaks could, of course, be attributed to denaturation of the lysozyme. However, two observations by Cousin et al. [47] speak against that: (i) Denaturation was only observed when the mixtures contained very large excesses of PSS (charge ratio = 20). For charge ratios ≈1, as in the shells, they claim that the protein maintained its native structure. (ii) When denaturation occurred, the SANS scattering both from the PSS and lysozyme was very weak in the whole *q*-range above 0.1 nm^−1^, and the lysozyme scattering displayed a quasi-plateau below *q* = 1 nm^−1^. In contrast, the scattering from our shells is strong at low *q*, with a *q*^−4^ decay law showing that there is good contrast between complexes and surroundings, and the scattering decreased in the entire *q*-range by more than three orders of magnitude. Furthermore, the scattering just above *q* = 1 nm^−1^ may be influenced by the form factor of the lysozyme, as indicated in Figure 7. The results are in agreement with a Fourier transform—infra red spectroscopy study by Wittemann and Ballauff indicating that the *α*-helix and *β*-sheet content of lysozyme was nearly fully retained when adsorbed to the PSS corona of spherical polyelectrolyte brushes [59]. Nevertheless, if the interactions between PSS and lysozyme responsible for the high density of the complexes are of hydrophobic nature they can be expected to have some effect on the conformation of the protein. In fact, Wu et al. [40] recently showed that nearly charge stoichiometric complexes of lysozyme and linear PSS contain a large fraction of lysozyme in a state different from the native form. In a mixture with a PSS/lysozyme-charge ratio of 1.5 at 20 °C, where the non-native state completely dominated, the contents of *α*-helix and *β*-sheet was reported to be 18% and 30%, as compared to 36% and 14%, respectively, in the native state. In conclusion, the SAXS profile at low *q* shows that the shell contains large but well-defined complexes of densely packed lysozyme molecules. The lysozyme is possibly in a non-native state, but most likely not completely unfolded. However, the features at higher *q* are difficult to interpret without detailed modelling. A schematic drawing of the shell microstructure is shown in Figure 8.

### 2.4. Cytochrome C Binding to PSS Gels

Cytochrome c (cyt c) is a protein of a size comparable to that of lysozyme and with a net charge of +5.8 at pH 8 [60]. It has been shown earlier that the two proteins behave differently with respect to binding with sodium polyacrylate (PA) networks. For example, while cyt c was found to diffuse more or less freely into weakly crosslinked PA microgels, causing them to contract considerably [22], lysozyme aggregated into a rigid shell that arrested the gel in a semi-swollen state [24]. Differences have also been observed when the proteins bind to PA macrogels [20], attributed to the presence of attractive protein–protein forces in the case of lysozyme and the absence of strong attractions between cyt c molecules [22]. To check if the difference remains for PSS networks, we carried out a minor study of the incorporation of cyt c into PSS macro- and microgels.

**Figure 9:**
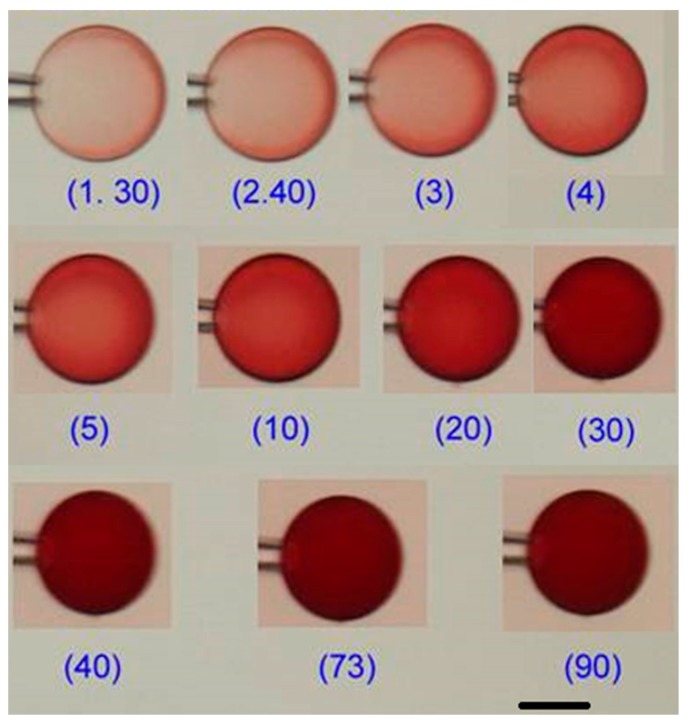
Microscopy images of PSS microsphere in a 729 ppm cytochrome solution (NaCl/tris buffer; pH 8.0, I = 10 mM) captured at different times in minutes as indicated. Scale bar = 50 µm.

Figure 9 shows pictures of cyt c binding to PSS microgels from a solution containing 729 mg/L of the protein; other conditions are the same as in Figure 3. As can be seen cyt c (red) diffuses rapidly towards the center. The diffusion front is blurred and no shell with a sharp boundary to the core can be observed at any time. The relative volume change is smaller than for lysozyme binding to gels A–D, but similar to that for gels E and F. This can be explained by a larger effective degree of crosslinking (*C*_10_ = 116 mM; *V*_0_/*V*_10_ = 1.05) than for the microgels used above, rather than by a difference between cyt c and lysozyme. Nevertheless, a clear difference between cyt c and lysozyme appears when looking at the time required for the volume relaxation, which is about one order of magnitude smaller for cyt c than for lysozyme. This suggests that the cyt c molecules diffuse more independently of each other in the network as compared to the lysozyme molecules. There is also another important difference. Unlike lysozyme, cyt c readily unbinds when the salt concentration in the liquid flowing around the gel is increased from 10 to 150 mM by addition of NaCl (Figure S5). This indicates that the interaction between PSS and cyt c is mainly electrostatic, as has previously been shown to be the case for the interaction between cyt c and PA. 

**Figure 10:**
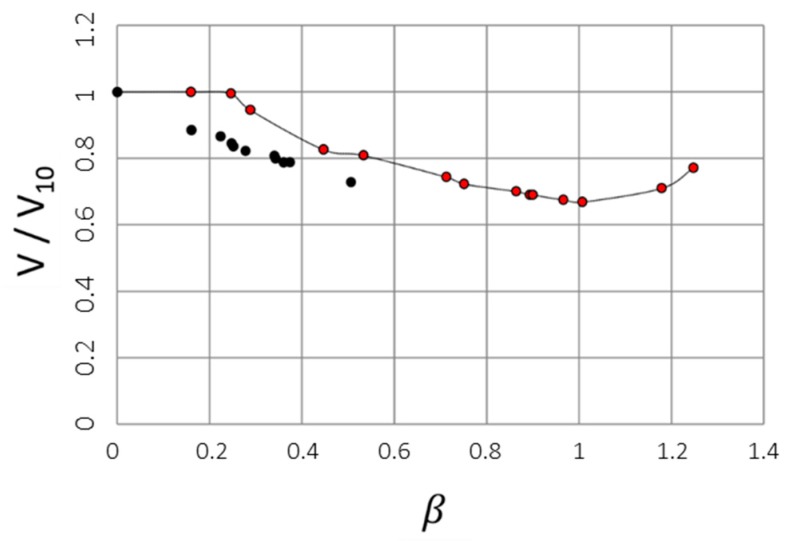
Volume ratio of PSS macrospheres as a function of binding ratio of lysozyme (black dots) and cytochrome c (red dots).

Figure 10 shows the equilibrium swelling (*V*/*V*_10_) of a PSS macrogel as a function of cyt c uptake (*β*). Data were recorded at the same conditions as for lysozyme/PSS macrogel in Figure 6 (both gels from the same batch; tris buffer, I = 10 mM, 500–1000 mg/L protein). The results for the latter system are included in Figure 10 for comparison. Obviously, at a given *β*, the incorporation of cyt c produces a smaller volume change than does lysozyme, a difference that is particularly striking for *β* < 0.25 where no volume change is seen for cyt c. Notable is also the reswelling observed with cyt c for *β* > 1. A minimum in gel volume at *β* ≈ 1 has previously been observed for cyt c/PA microgels and the behavior has been rationalized by theoretical model calculations [61,62]. From the volume ratio at *β* = 1 one can observe that the concentration of PSS charges and the equivalent concentration of cyt c net charges in the gel is equal to 123 mM and the water content is 80 wt %. A comparison with the corresponding values (184 mM PSS charges and 60 wt % water) for the shells formed by lysozyme (*f_s_* = 0.97) in the same type of gel shows that lysozyme form denser complexes. A comparison with data for PA macrogels is also illuminating. Information is only available for mixtures without added salt. However, in that case the water contents are 75 and 44 wt % for cyt c and lysozyme, respectively [16,17,18,20]. The complexes are less swollen, as expected in the absence of salt, but the difference between the two proteins is nevertheless similar to that in the PSS case. We conclude that the difference between the two proteins is present both in the PSS and PA systems and that they seem to derive from differences in the protein–protein interaction rather than the protein–polyion interaction.

In another experiment cyt c was used to probe the permeability of shells formed by lysozyme. A lysozyme shell was first formed in a spherical PSS macrogel. After that, the gel was immersed in a solution of cyt c. After some time, when the gel had acquired a red colour, a 1 mm thick slice through the middle of the gel was removed and analyzed in the microscope. The result was very clear: cyt c had diffused through the shell to the core (see Figure S6 in the Supplementary Material S1) and the shell remained intact. Earlier, Karabanova et al. [17] performed the same type of experiment with PA gels, but with a different result. They reported that cyt c formed a shell outside the preformed lysozyme shell, thereby “pushing” the latter inwards without exchanging molecules between layers, a mechanism proposed to be general for the binding of macroions [16,20,25]. In conflict with that (but in agreement with what we observe here) fluorescently-labelled lysozyme has been shown to diffuse through the preformed lysozyme shell in PA microgels [22]. A similar behaviour has also been observed when proteins are incorporated into non-responding ion exchange matrices [63] and in other related systems [64]. 

### 2.5. Role of Hydrophobic Interactions

Lysozyme is well-known for its self-aggregating properties. Depending on the conditions it can form finite size aggregates [65], 3-dimensional (3-d) networks, glassy states [37], and crystals [66]. A minimalistic yet powerful model employed to account for these observations is a system of charged spheres (or globules) interacting via long range electrostatic repulsions and short range (non-electrostatic) attractions. In such a model, aggregation will take place in environments providing sufficient screening of the electrostatic repulsions. From the work by Cousin, Gummel and co-workers [10,46,47,48,49] it is clear that PSS chains can provide such an environment, and that it preferentially leads to the formation of large (but finite size) complexes comprising nearly charge-equivalent amounts of lysozyme and polyion chains. The SAXS results presented above show that the molecules are arranged in a similar way in shells formed when lysozyme binds to PSS gels. The stability of the complexes even at high salt concentration shows that non-electrostatic, presumably hydrophobic, interactions are important. Thus, we believe that the shell is to a large extent built up from aggregated lysozyme, and that the aggregates are stabilized both by electrostatic and hydrophobic interactions with the PSS chains and by hydrophobic protein–protein attractions. The importance of the hydrophobic interaction is highlighted also by the fact that cyt c, a molecule known not to possess strong non-electrostatic interactions [38], does not form dense complexes or aggregates in the gel.

### 2.6. Binding Mechanism

The microscopy images indicate that a core–shell morphology is created and maintained during the course of lysozyme binding to the PSS microgels. After an initial period of substantial volume decrease there is an extended period of minor volume decrease. During the latter period the core–shell boundary continues to move toward the centre and the shell appears to become inhomogeneous. Interestingly, the rate of the moving boundary and the inhomogeneity of the shells increase with decreasing (apparent) degree of crosslinking. To account for these observations we propose that during the first period a fairly homogeneous shell is formed. The process can be described as a frontal heterogeneous reaction [16], in which the collapsed shell grows at the expense of the swollen core. The volume change of the gel is directly related to the difference in swelling between the two phases. The very slow dynamics of the process suggest that the mass transport is hindered by energetic and/or kinetic barriers. An electrostatic energy barrier would be present if the ratio between network and protein charges in the shell is below unity ($f_{s}<1$). It could hinder protein molecules from entering the gel from the solution, but could also exclude network counterions from the shell, thereby affecting the transport of counterions out from the core, which is necessary for the inward motion of proteins (ion exchange). Aggregation and/or complexation of the protein with the network and the formation of highly dense microstructures would severely affect the mobility of the protein. In an idealized case there are two states of the protein in the shell: Mobile molecules and stationary molecules bound to aggregates or complexes. If the exchange of molecules between the two states is slow the transport rate should depend on the concentration gradient(s) of the mobile species in the shell and their diffusion coefficient(s). A combination of obstruction effects and low free concentration in the shell could thus lead to low permeability. If the exchange is fast, the transport should be similar to collective diffusion driven by the chemical potential gradient in the shell. Here the effective diffusion coefficient would be determined by the fraction of free molecules and their diffusion coefficient. If the local equilibrium is such that the free fraction is very low, the result would likewise be low permeability.

Another requirement for volume change during shell growth is that the shell must be able to adapt to the shrinking core. If the shell adapts slowly, the process may become rate limiting. Important here is that each volume element in the shell moves closer to the gel centre and changes shape as the gel volume decreases [67,68]. This requires rearrangements of the molecules both on the local and global scale. Reasons for slow adaptation can be jamming of the complexes or simply that the network chains and the proteins are restricted in their motions relative to each other by the intimate interactions in the complexes. Our hypothesis is that the relaxation of the shell becomes so slow that the volume change practically stops when the protein molecules building up the shell reach a critical number. The shell is still permeable to protein molecules (as indicated by the penetration study), but due to its rigidity the growth is forced to propagate without collapsing the gel network to the same extent as before. We believe this marks the onset of the second period, in which the decrease of the gel volume is minor, but a sharp diffusion front continues to migrate inward. To explain why the rate of the diffusion front during this period increases with the decreasing (apparent) degree of crosslinking, we further propose that the protein molecules become incorporated at a lower packing density than in the outer parts of the shell, and in particular that the packing density is lower, the lower the polyelectrolyte concentration in the core. The motive behind the latter would be to maintain the ratio between network and protein charges close to unity. However, at the same time, it follows from geometry that the thickness of the dense outer part of the shell decreases with increasing swelling of the core network, and therefore that the mass transport rate through it increases. Thus, if the transport through the outer shell layer is rate controlling, the rate of the core diffusion front should decrease with decreasing degree of crosslinking, in agreement with the experiments. To test this idea we employed a simple model for the consecutive build-up of the outer and inner layers of the shell in a spherical gel, denoted shell (*s*) and core diffusion layer (*cdl*), respectively. The model combines the geometrical description behind Equation (5) with a description of protein mass transport from the liquid solution, through both layers, to the core diffusion front at the protein-free core. A derivation of the model equations is given in the Supplementary Material S4. The rate of binding is described by the following relationship valid at steady state:

(8) $${\left(\frac{d\beta }{dt}\right)}_{ss}=\frac{3ZP_{cdl}P_{s}{\bar{r}}_{0}{\bar{r}}_{1}{\bar{r}}_{2}C_{2}}{C_{p0}R_{0}^{2}\left\{P_{s}{\bar{r}}_{2}\left({\bar{r}}_{1}-{\bar{r}}_{0}\right)+P_{cdl}{\bar{r}}_{0}\left({\bar{r}}_{2}-{\bar{r}}_{1}\right)\right\}},$$

$C_{2}$ is the protein concentration in the liquid, $C_{p0}$ is the concentration of network charges in the protein-free gel, $P_{cdl}$ is the permeability of the core diffusion layer and $P_{s}$ is the permeability of the outer shell. The scaled quantities ${\bar{r}}_{n}\equiv r_{n}/R_{0}$, with $r_{0}$ being the radius of the protein-free core, $r_{1}$ the position of the boundary between the inner and outer shells, and $r_{2}$ the gel boundary, are related to *β*:

(9) $${\bar{r}}_{0}^{3}=1-f_{s}\beta_{s}-f_{cdl}\left(\beta -\beta_{s}\right),$$

(10) $${\bar{r}}_{1}^{3}=1-f_{s}\beta_{s},$$

(11) $${\bar{r}}_{2}^{3}=1-f_{s}\beta_{s}+{\bar{v}}_{s}f_{s}\beta_{s}.$$

Here ${\bar{v}}_{s}\equiv v_{s}/v_{0}$, where $v_{s}$ is the volume per network charge in the outer shell, $f_{cdl}$ and $f_{s}$ are the ratio between network and protein charges in the inner and outer shell, respectively, and $\beta_{s}$ is the number of protein charges in the outer shell per network charges in the entire gel. The above relationships are valid when the swelling of the network in the protein-free core and the core diffusion layer both are equal to that of the network in the protein free gel ($v_{0}=v_{c}=v_{cdl}$; ${\bar{v}}_{c}={\bar{v}}_{cdl}=1$). Then, since ${\bar{r}}_{2}^{3}=V/V_{0}$ and ${\bar{r}}_{1}^{3}={\bar{r}}_{2}^{3}-\frac{V_{s}}{V_{0}}={\bar{r}}_{2}^{3}-{\bar{v}}_{s}f_{s}\beta_{s}$, Equations (10) and (11) follow directly from Equation (5). Furthermore, since all protein molecules in the gel are located in the shell and the core diffusion layer we can write ${\bar{r}}_{1}^{3}={\bar{r}}_{0}^{3}+f_{cdl}\left(\beta -\beta_{s}\right)$. This relationship used in Equation (10) gives Equation (9).

The neglect of stagnant layer diffusion is motivated by the very slow uptake indicating that the process is governed by particle diffusion control. By using Equations (9)–(11), Equation (8) can be integrated to give a relationship between *t* and *β*, from which in turn relationships between *t* and, respectively, ${\bar{r}}_{0}$, ${\bar{r}}_{1}$, and ${\bar{r}}_{2}$ can be obtained. From the latter set the evolution of the volumes of the protein-free core and the gel are easy to calculate. The growth of the outer shell is considered to end abruptly at a given *β*′, after which no further deswelling and growth takes place. Thus, integration is made in two steps according to:

(12) $$\beta \leq \beta^{\prime }:\;\;\;\;\;\;\{\begin{array}{c} {\bar{r}}_{0}={\bar{r}}_{1}\\ \beta =\beta_{s} \end{array},$$

(13) $$\beta >\beta^{\prime }:\;\;\;\;\;\{\begin{array}{c} {\bar{r}}_{1}={\bar{r}}_{1}\left(\beta^{\prime }\right)\\ {\bar{r}}_{2}={\bar{r}}_{2}\left(\beta^{\prime }\right)\\ \beta_{s}=\beta^{\prime } \end{array}$$

For each of the systems A–F we used the data in Table 1 according to $R_{0}=R_{10}$, $C_{p0}=C_{10}$, $C_{2}=C_{lys}/M$ (M = 14,300 g/mol). All other parameters have the same value for all systems: $P_{cdl}=6\times 10^{-10}\;m^{2}/s$;$\;P_{s}=7.5\times 10^{-13}{\;m}^{2}/s$; Z=7.6; $f_{cdl}=f_{s}=1$; $\beta_{s}\left(t^{\prime }\right)=0.5$, $v_{s}$= 0.0054 m^3^/mol. The result is shown in Figure 5c,d. The most interesting feature is that the model captures qualitatively the differences in the core diffusion rates between the gel species as observed in the experiments (Figure 5a,b). The calculated rate depends strongly on the permeability assigned to the outer shell, but is insensitive to the permeability of the inner shell, showing that the transport through the former is rate controlling. In fact, the variation between the systems is to a large extent determined by the difference in the final thickness of the outer shell layers, which according to the model calculations are 2.8 (A), 4.0 (B), 8.45.4 (C), 4.2 (D), 5.6 (E), and 5.3 (F) µm. $P_{cdl}$ is equal to the diffusion coefficient of free lysozyme in water [69]. The values of $\beta_{s}\left(t^{\prime }\right)$ and $P_{s}$ were adjusted to give reasonable agreement with the magnitude and the time scale of the gel volume change, respectively. The value of the latter is quite realistic considering the factors determining the permeability discussed above.

## 3. Conclusions

The interaction between lysozyme and PSS gels leads to the formation of dense complexes between the protein and the network chains. During the dynamic process of binding from solution, the complexes form a shell in the outer layers of spherical gels. Investigations of macrogels show that the shell contains 60 wt % water and nearly charge equivalent amounts of polyion and protein arranged in finite size clusters (diameter = 16 nm) comprising 30–60 protein molecules. The growth of the shell and the accompanying gel volume decrease are very slow. For microgels, the process can roughly be divided into three sub-processes, the distinction between which becomes clearer the lower the degree of crosslinking of the gel network. During the first, a dense apparently homogeneous shell is formed. During the second, the shell grows with lower protein density, and ends when the (sharp) protein diffusion front reaches the gel center. With support from the kinetic modelling results, we propose that the rate of the diffusion front is controlled by mass transport through the dense outer part of the shell. During the third period, which was not studied in detail, the heterogeneous gel relaxes slowly, presumably towards a compact homogeneous equilibrium state.

The aggregates in the gels are stabilized both by hydrophobic and electrostatic interactions, where attractive protein–protein interactions play an important role. In the absence of interactions of the latter type, as exemplified by cyt c, the binding mechanism is qualitatively different and characterized by fast diffusion of protein in the gel without a sharp core/shell boundary. The behavior of lysozyme in the most weakly crosslinked PSS microgel resembles that of lysozyme in PA microgels, but the overall process is an order of magnitude slower. The very slow dynamic is a novel feature that we attribute to the combination of electrostatic and hydrophobic interactions between the protein and the PSS network and hydrophobic protein–protein interactions. Lysozyme has been used in many scientific investigations as a model protein displaying self-assembling and aggregation properties. The behavior described here is therefore expected to be generic for self-assembling proteins interacting with oppositely-charged polymer networks made up of chains with hydrophobic backbones. The results should be useful in applied areas such as protein and peptide drug formulation.

## 4. Materials and Methods

### 4.1. Chemicals

Sodium poly(styrene sulfonate) (NaPSS) (completely sulfonated, *M*w = 70,000) from Polysciences, sodium styrene sulfonate hydrate (NaSS·H_2_O) from Aldrich (Saint Louis, MO, USA), paraffin oil from VWR International, NaCl from Fisher Chemical, Silicone Oil from Fluka, and tris(hydroxymethyl)aminomethane, ammonium persulfate (APS), *N*,*N*,*N*′,*N*′-tetramethylethylene-diamine (TEMED), and *N*,*N*′-methylene-bis-acrylamide (BIS) from Sigma-Aldrich (Steinheim, Germany), were all used as received. Lysozyme (from chicken egg white, ≥95%) from Sigma-Aldrich (Germany) and cytochrome c from horse heart from Fluka BioChemie (Fluka A.G., Buchs, Switzerland) were dissolved in water and dialyzed to remove excess of simple salts, filtered (0.22 µm) and lyophilized prior to use. All solutions were prepared with Millipore water.

### 4.2. Preparation and Characterization of PSS Gels

#### 4.2.1. Macrogels

Gels were synthesized by free radical polymerization (FRAP) [70]. Spherical gels were made by injecting a reaction mixture (150 µL) into a density matched medium consisting of a mixture of silicone oils (*ρ* = 1.06–1.09 g/mL) contained in Eppendorph tubes (one for each gel particle). The tubes were sealed and stored overnight at 65 °C. The reaction mixture had the following composition: 7.5 g NaSS, 0.75 g BIS, 30 g water, 72 µL TEMED and 1.350 mL of 0.18 M APS-solution. Gel slabs (macrogels) of different size (dry weight 0.002–0.03 g) were synthesized in the presence of various amounts of crosslinker (8–10 wt %). After synthesis the gels were washed in pure water, dried at room temperature and weighed. 

#### 4.2.2. Microgels

Spherical particles of cross-linked poly(styrene sulfonate) were synthesized by free radical polymerization in a w/o suspension in an RB-flask containing paraffin oil. Before use, the oil was preheated to 98 °C and bubbled with N_2_ for 4 h to remove oxygen. The temperature was thereafter set to 65 °C and the reaction solution was added slowly under magnetic stirring (1000 rpm). In total, 1.5 mL was added to the oil, 1–2 min before the estimated time of gelation. Gel particles were extracted from the oil after 30 min. The product was a collection of spherical microgels with a broad size range. Particles were then washed and stored in pure water. 

#### 4.2.3. Characterization

The responsiveness of gel networks was determined by measuring their equilibrium volumes at free swelling in aqueous NaCl solutions (0–150 mM). For microgels this was done by measuring the diameter in the microscope set-up prior to the protein experiment. The PSS concentration in a macrogel in equilibrium with pure water was determined gravimetrically by weighing the gel before and drying, first in a freeze dryer for 48 h (Flexidry µP freeze-dryer, Kinetics Thermas Systems, Stone Ridge, NY, USA), and thereafter at 105 °C until no further weight loss could be observed (24 h). The concentration of macrogels in NaCl solutions was calculated from measurements of the volume change relative that in pure water.

### 4.3. Protein Binding to Macrogels

Macroscopic gel spheres where placed in 500–1000 mg/L protein solutions in Tris buffer (pH 8.0). The solutions contained 5 mM NaCl and the total ionic strength was 10 mM. After equilibration in sealed containers for seven months at 22 °C the concentration of protein in the liquid was determined spectrophotometrically, for lysozyme at 282 nm (*ε* = 3.62 × 10^4^ M^−1^·cm^−1^), and for cytochrome c at 408 nm (*ε* = 1.10 × 10^5^ M^−1^·cm^−1^). All equipment was sterilized and solutions were filtered (0.22 µm) prior to the study.

### 4.4. Protein Binding to Microgels

Microgels were studied using a light/fluorescence microscope (Olympus BX-51, Olympus, Tokyo, Japan) equipped with a micromanipulator (Narishige ONM-1, Narishige, Tokyo, Japan), digital camera (Olympus DP50, Olympus, Tokyo, Japan), software (Olympus DP-Soft, Olympus, Tokyo, Japan), and OlympusU-RFL-T UVlamp (Olympus, Tokyo, Japan). Lysozyme auto-fluorescence was monitored at wavelengths >400 nm. Microcapillaries were prepared and polished using a Narishige PC-10 Puller and a MF-9 Forger. Microgels were picked up with the micropipette by suction, using an IM-5A Injector (Narishige, Tokyo, Japan). The volume of a microgel was determined from the diameter assuming a spherical shape. Single microgels were studied under conditions of forced convection by positioning them in a liquid flow inside a glass flow tube (diameter 1.4 mm) by means of the micromanipulator/micropipette, as described in detail elsewhere [43,50,71]. A pump was used to feed the fresh protein solution from a reservoir to the flow tube. To obtain a slow but steady flow around the microgel, a container, acting as a damper, was positioned between the pump and the flow tube. The flow rate was controlled by adjusting the height of the liquid level in the container by fine-tuning with the aid of a small elevator to match the volume flow provided by the pump. The setup enabled a steady flow rate at the centre of the capillary (6 mm/s) for extended periods of time. In a typical experiment, a microgel was first transferred from a 10 mM NaCl storage solution to the flow tube and exposed to aqueous solutions of NaCl of different concentrations. The volume response was used as a characteristic of the responsiveness of the microgel network to osmotic stress. After that the microgel was pre-equilibrated in NaCl/tris buffer (pH 8.0, I = 10) and then exposed to the protein solution in the same medium.

### 4.5. Small-Angle X-ray Scattering

SAXS measurements were performed at the Dutch-Flemish CRG beam line (BM26B) at the European synchrotron radiation facility (ESRF) in Grenoble, France. The SAXS intensity is expressed as a function of the scattering vector modulus, q=(4π/λ)sin(θ/2), where λ is the wavelength of the X-rays (1Å) and θ is the scattering angle.

## Figures and Tables

**Table 1:** Description of the investigated lysozyme–microgel systems.

Gel Particle	*R*_10_ (µm) ^a^	*V*_0_/*V*_10_^b^	*C*_10_ (mM) ^c^	*C*_lys_ (mg/L) ^d^	*V_β_*_=1_/*V*_10_^e^
A	49	2.0	43	800	0.23
B	55	1.7	55	800	0.30
C	62	1.5	67	800	0.36
D	49	1.5	67	500	0.36
E	59	1.4	74	500	0.40
F	56	1.4	74	500	0.40

^a^ Microgel radius in 10 mM NaCl (no protein); ^b^ Ratio of microgel volume in pure water and in 10 mM NaCl; ^c^ Concentration of network charges in microgel in 10 mM NaCl; ^d^ Concentration of lysozyme in solution in contact with microgel; ^e^ Theoretically calculated ratio of volume of microgel with lysozyme-to-PSS charge ratio equal to unity and protein-free gel in 10 mM NaCl, assuming that the microgels are homogeneous with the same composition as the shell of macrogel.

## Supplementary Materials

The following are available online at www.mdpi.com/2310-2861/4/1/9/s1, Figure S1: Light microscopy images of microgel A at different times during deswelling in 800 mg/L lysozyme solutions (NaCl/tris buffer; pH 8.0, I = 10 mM). UV-vis: illumination by mixture of UV and and visible light. Inset: Gel A in equilibrium with protein-free electrolyte solutions of ionic strengths 0 and 10 mM, Figure S2: Light microscopy images of microgel B at different times during deswelling in 800 mg/L lysozyme solutions (NaCl/tris buffer; pH 8.0, I = 10 mM). Inset: Gel B in equilibrium with protein-free solutions NaCl solutions of various concentrations. “*β* = 0.55”: Macrogel with lysozyme-rich shell, Figure S3: Microgel C at different times during deswelling in 800 mg/L lysozyme solutions (NaCl/tris buffer; pH 8.0, I = 10 mM). Lower row: Gel C after 24 h in the protein solution, after flushing with 20 mM SDS for 5–10 s, 170 s, and 5 min (fluorescence microscopy image). Left inset: Gel C in equilibrium with protein-free NaCl solutions of various concentrations, Figure S4: Microgel E at different times during deswelling in 500 mg/L lysozyme solutions (NaCl/tris buffer; pH 8.0, I = 10 mM). Two left-most columns: Gel E in equilibrium with protein-free NaCl solutions of various concentrations prior to measurement. Right: Core-shell formation observed with different optical settings with or without fluorescent light. Volume decrease is observed upon reduction of pH, Figure S5: Time resolved volume decrease of a PSS microsphere in a cytochrome c solution of 729 ppm and in the standard, Figure S6: Pictures of a PSS macrosphere sliced apart to display a 1 mm thick centerpiece after consecutive binding of lysozyme and cyt c. The rightmost image shows that cyt c (red) has diffused through the lysozyme rich surface phase. The core spontaneously detached from the shell after additional incubation of the slice in a closed container, possibly the result of the stress from cyt c induced contraction of the core, Figure S7: The concentration of network charges in NaPSS macrogels in 10 mM NaCl solution (*C*_10_) as a function of the ratio of the gel volume in pure water (*V*_0_) and in 10 mM NaCl (*V*_10_). Symbols: Experimental data. Dashed line: Equation (S1) fitted to the experimental data. Solid line: Theoretical curve calculated from Equations (S2) and (S3) with x = 16, 𝛼 = 0.35 and *C** = 2 M, Figure S8: Volume (*V*) of microgel in NaCl solution relative that in pure water (*V*_0_) plotted vs. the concentration of NaCl in the solution (*C*_salt_). Symbols: Experimental data. Curves: Theoretical model calculated with *x* = 16, 𝛼 = 0.35, C* = 2 M; *s* = 15 (A), 11 (B), 8.7 (C), 8.4 (D), and 6.7 (E,F), Figure S9: Logarithm of the scattering intensity in the low *q*-range (ln I) plotted vs. the square of the scattering vector (*q*^2^) (Guinier plot). Data from synchrotron SAXS experiments on the shells formed in PSS macrogels after absorbing lysozyme from solution.
