# Supplementary material for: Binding of Lysozyme to Spherical Poly(styrenesulfonate) Gels
Source: Gels. 2018 Jan 16;4(1):9. doi: 10.3390/gels4010009 (PMC6318605; doi:10.3390/gels4010009)
Supplement: Supplementary file 1 [file gels-04-00009-s001.pdf]

Supplementary Material to the Article

Binding of Lysozyme to Spherical Poly(styrenesulfonate) Gels

Martin Andersson and Per Hansson

Department of Pharmacy, Box 580, SE-75123 Uppsala University, Sweden

S1. Microscopy Images of Microgels

Pictures of microgels A, B, and C at various stages during the binding process in 800 mg/L lysozyme are displayed in Figures S1, S2, and S3, respectively. Figure S4 shows the behavior of microgel E in 500 mg/L lysozyme solutions. Shown are also the same particles in protein-free solutions at various ionic strengths. Fig. S4 shows pictures of a PSS microgel interacting with cytochrome c (cyt c), and Fig. S5 shows pictures of PSS macrogel with a pre-formed lysozyme rich shell after the subsequent absorption of cyt c.

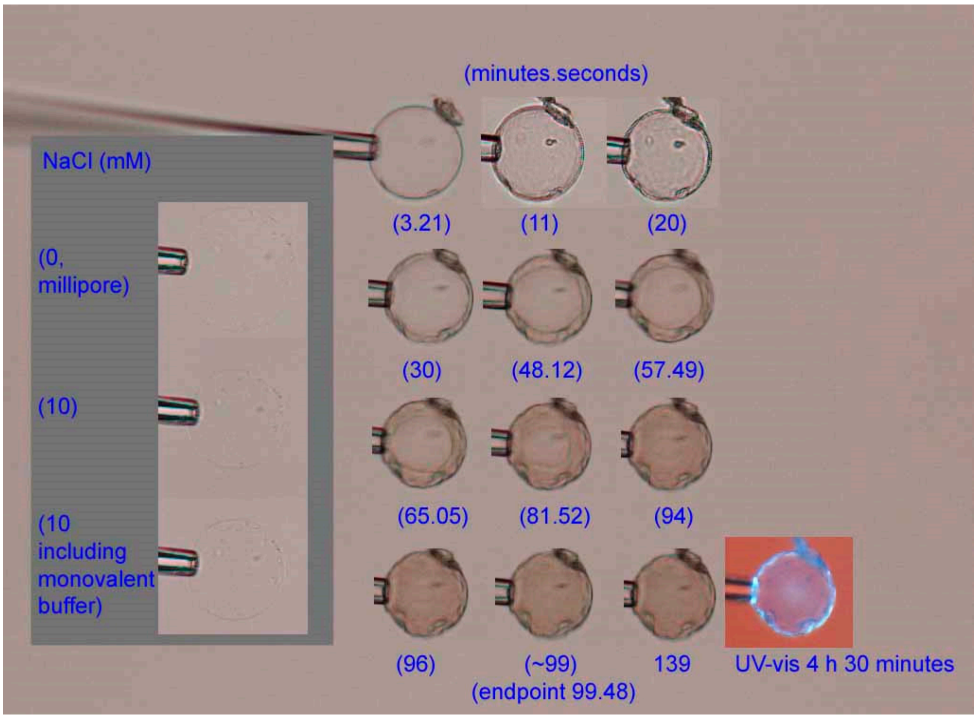

Figure S1. Light microscopy images of microgel A at different times during deswelling in 800 mg/L lysozyme solutions (NaCl/tris buffer; pH 8.0, I=10 mM). UV-vis: illumination by mixture of UV and and visible light. Inset: Gel A in equilibrium with protein-free electrolyte solutions of ionic strengths 0 and 10 mM.

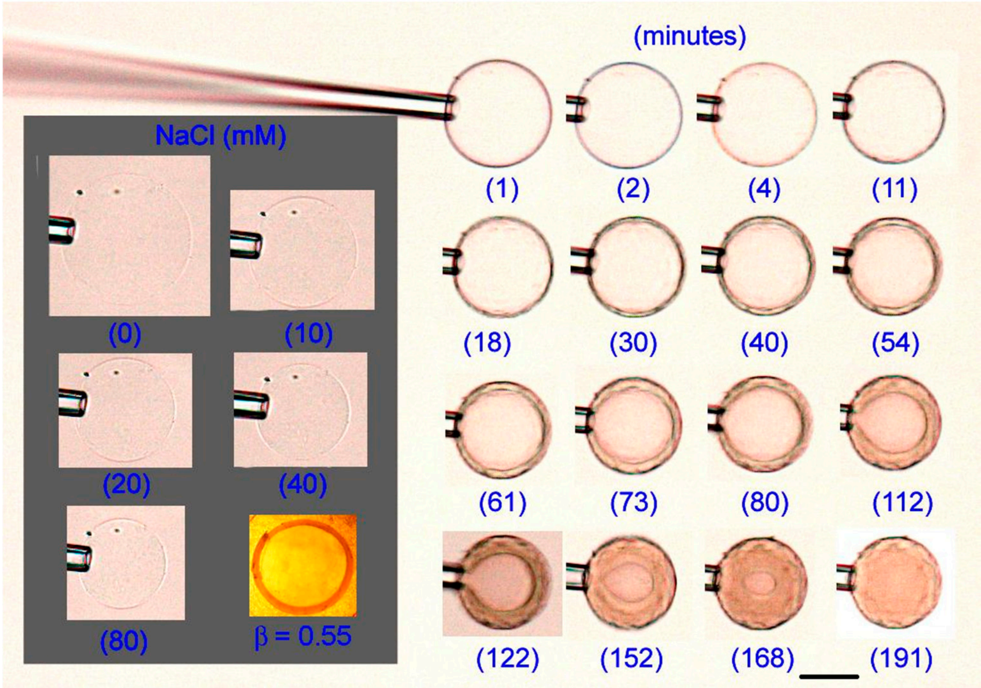

Figure S2. Light microscopy images of microgel B at different times during deswelling in 800 mg/L lysozyme solutions (NaCl/tris buffer; pH 8.0, I=10 mM); scale bar = 50 μm. Inset: Gel B in equilibrium with protein-free solutions NaCl solutions of various concentrations. "β=0.55": Macrogel with lysozyme-rich shell.

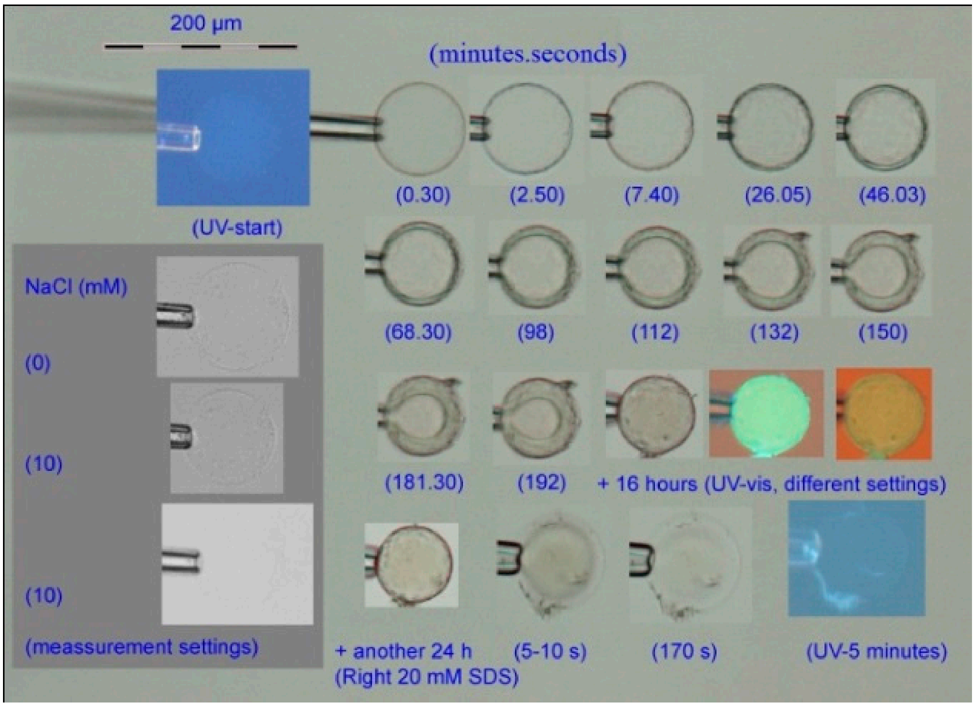

Figure S3. Microgel C at different times during deswelling in 800 mg/L lysozyme solutions (NaCl/tris buffer; pH 8.0, I=10 mM). Lower row: Gel C after 24 h in the protein solution, after flushing with 20 mM SDS for 5-10 s, 170 s, and 5 minutes (fluorescence microscopy image). Left inset: Gel C in equilibrium with protein-free NaCl solutions of various concentrations.

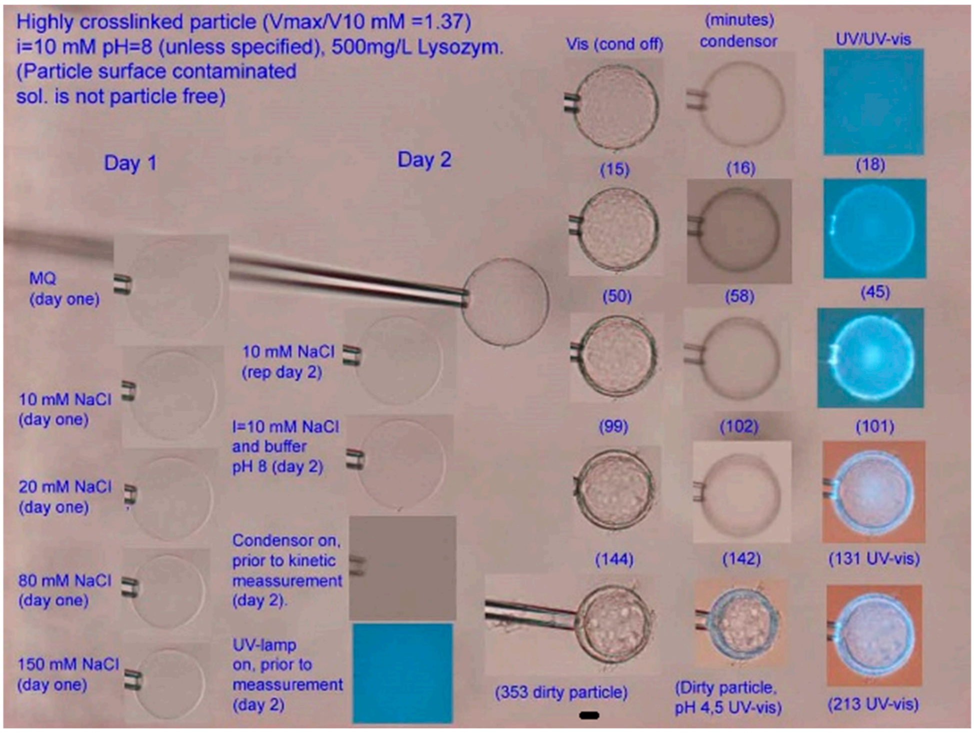

Figure S4. Microgel E at different times during deswelling in 500 mg/L lysozyme solutions (NaCl/tris buffer; pH 8.0, I=10 mM). Two left-most columns: Gel E in equilibrium with protein-free NaCl solutions of various concentrations prior to measurement. Right: Core-shell formation observed with different optical settings with or without fluorescent light. Volume decrease is observed upon reduction of pH. Scale bar = 25 µm.

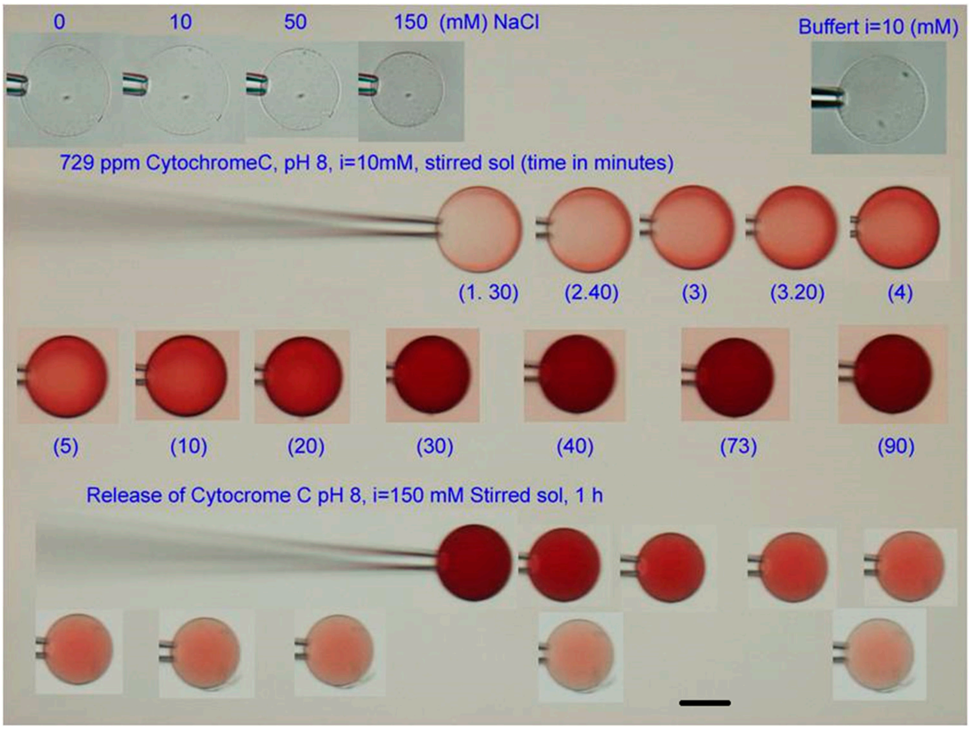

Figure S5. Time resolved volume decrease of a PSS microsphere in a cytochrome solution of 729 ppm and in the standard. Scale bar = 50 µm.

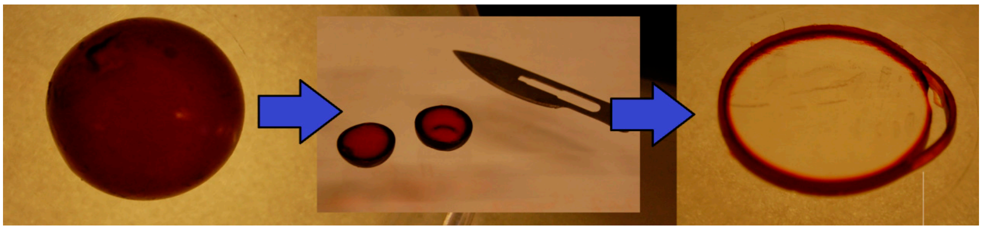

Figure S6. Pictures of a PSS macrosphere sliced apart to display a 1 mm thick centerpiece afer consecutive binding of lysozyme and cyt c. The rightmost image shows that cyt c (red) has diffused through the lysozyme rich surface phase. The core spontaneously detached from the shell after additional incubation of the slice in a closed container, possibly the result of the stress from cyt c-induced contraction of the core.

S2. Application of the scaling law for the swelling of macrogels to microgels.

In the Article the concentration of network charges in PSS microgels at swelling equilibrium in 10 mM NaCl solution (C_{10}) is calculated using the empirical scaling law for spherical PSS macrogels (diameter ~1 cm) given in Fig. 2 in the Article:

$$\frac{C_{10}}{mM} = 125 \times \left(\frac{V_0}{V_{10}}\right)^{-1.552} \qquad \text{(S1)}$$

with V_0 and V_{10} being the volume in pure (milli-q) water (C_0) and in 10 mM NaCl, respectively. In the thermodynamic sense, the present microgels are of macroscopic size, and so eq. (S1) should be applicable to them as long as they have the same type of network structure as the the macrogels. The data in Figure 1b in the Article strongly suggest that this is the case for microgel B. In that case the comparison is straightforward since, by coincidence, microgel B has nearly the same V_0/V_{10} value as one of the macrogel batches. For the other micogels this is not the case. To justify the validity of eq. (S1) for these microgels we show that a theoretical model that gives a reasonable description of eq. (S1) in the relevant range of V_0/V_{10} –values can also be fitted to the salt response curves for all the microgels.

The model takes into account the contributions to the free energy from elastic deformation of the network and the entropy of mixing. The former is described using the inverse Langevin theory of rubber elasticity from which the contribution to the osmotic pressure of a uniformly swollen network is [1,29]:

$$\frac{\Pi^{def}}{RT} = \frac{C}{xs}\left(\frac{1}{2} - \left(\frac{C^*}{C}\right)^{\frac{2}{3}} - \frac{3}{5s}\left(\frac{C^*}{C}\right)^{\frac{4}{3}} - \frac{99}{175s^2}\left(\frac{C^*}{C}\right)^{2} - \frac{513}{875s^4}\left(\frac{C^*}{C}\right)^{\frac{8}{3}} - \cdots\right) \qquad \text{(S2)}$$

Here, R is the ideal gas constant, T is the absolute temperature, C is the concentration of styrene sulfonate (ss) units in the swollen gel, C^* is the corresponding concentration in a relaxed reference state, x is the number of ss units in a statistical segment and s is the number of statistical segment between cross-links. The contribution from the entropy of mixing of the mobile ions to the osmotic pressure difference between the gel and the salt solution is calculated as:

$$\frac{\Delta\Pi^{ion}}{RT} = ((C\alpha)^2 + 4C_{salt}^2)^{\frac{1}{2}} - 2C_{salt} \tag{S3}$$

Here, C_{salt} is the concentration of monovalent salt in the solution. Eq. (S3) takes into account the partitioning of salt between the gel and the solution and the effect of counterion binding to the network. The latter effect is handled in a simple way by assuming that a constant fraction α of the network charges are dissociated from their counterions. The dissociated counterions and the salt ions that have entered from the solution are treated as freely mobile in the gel. The contributions to the osmotic pressure from the non-electrostatic interactions between the network chains and from the entropy of mixing the network and water are neglected, as justified by the swollen state of the network and the dominating contribution from eq. (S3) in gels with highly charged networks.

Equilibrium swelling is obtained when the condition $\Pi^{def} + \Delta\Pi^{ion} = 0$ is fulfilled. Figure S7 shows a fit of the model to eq. (S1). The curve was obtained by calculating C_0 and C_{10} as a functions of s with the parameters x, $C^*$and α as fitting parameters, and using the relationship $\frac{V_0}{V_{10}} = \frac{C_{10}}{C_0}$. The *shape* of the curve is largely independent on the parameters but the *position* in the vertical direction is very sensitive to α; it is less sensitive to x and practically independent on C^*. Clearly the model does not reproduce the linear scaling law but the agreement is quite good in the interval covering the swelling amplitudes of microgels A – F (marked by the arrows in the figure).

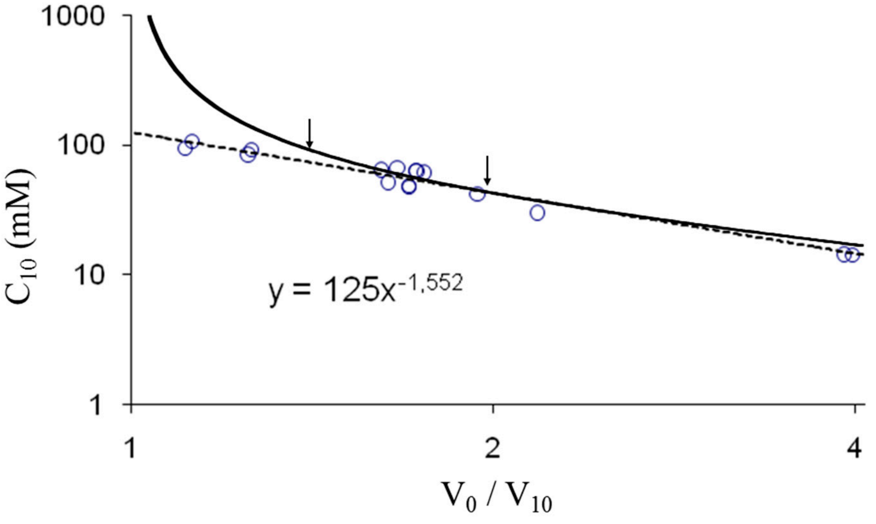

Figure S7. The concentration of network charges in NaPSS macrogels in 10 mM NaCl solution (C_{10}) as a function of the ratio of the gel volume in pure water (V_0) and in 10 mM NaCl (V_{10}). Symbols: Experimental data. Dashed line: Eq. (S1) fitted to the experimental data. Solid line: Theoretical curve calculated from eqs. (S2) and (S3) with x=16, α=0.35 and C^*=2 M.

The values of the parameters obtained from the fit are quite reasonable for the present type of network. There is a covariance in such a way that the value of α required to make the fit good in the relevant interval increases with increasing x. The highest possible α-value, corresponding to x=1, is 0.60, showing that, within the model, a certain degree of counterion binding to the network is necessary. With x=16 one obtains α=0.35, a value equal to the inverse of the linear

charge density parameter (ξ) for PSS in the Osawa-Manning counterion condensation theory. This equals the inverse of the apparent degree of dissociation of the polyelectrolyte in the simplest version of that theory, in agreement with the above definition of α. Furthermore, a Kuhn statistical segment of 16 ss units, corresponding roughly to a persistent length of 2 nm, is not at all unphysical. Figure S8 shows fits of the model to the salt response curves for microgels A – F in the relevant lower range of salt concentrations. The curves were obtained by adjusting the parameters s and C^*, keeping the set {x=16; α=0.35} fixed. Again there is covariance between the parameters so that different s - C^* pairs yield identical fits. The displayed curves are valid for C^*=2 M and the following s-values: 15 (A), 11 (B), 8.7 (C), 8.4 (D), and 6.7 (E, F). The value of C* is arbitrarily selected, of course, but quite reasonable for the present gels considering the concentration of ss in the reaction mixture (1.2 M). The resulting number of ss units between crosslinks (=xs) is: 238 (A), 176 (B), 139 (C), 134 (D), and 107 (E, F).

In conclusion, the theoretical model provides a reasonable description of the macrogel data in the range of V_0/V_{10} displayed by the microgels. The same model, with the parameters determined for the *macrogels* fixed, can also be fitted to independent swelling data for the *microgels*. The result suggests a similarity between the networks in the macrogels and the microgels.

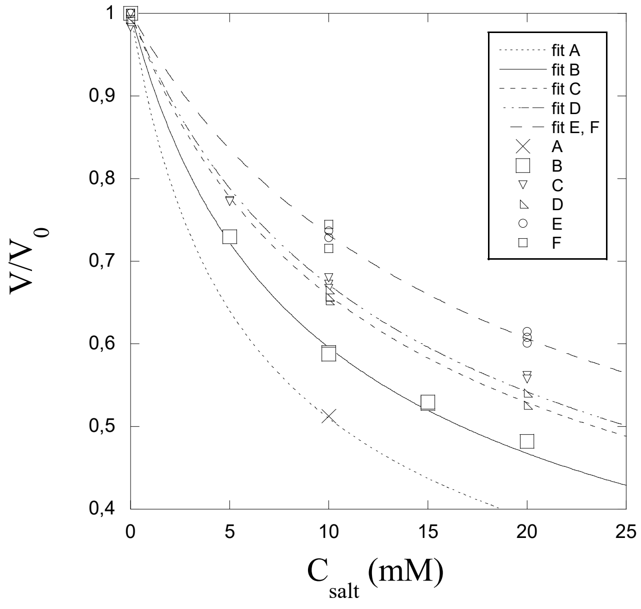

Figure S8. Volume (V) of microgel in NaCl solution relative that in pure water (V_0) plotted vs. the concentration of NaCl in the solution (C_{salt}). Symbols: Experimental data. Curves: Theoretical model calculated with x=16, α=0.35, C^*=2 M; s = 15 (A), 11 (B), 8.7 (C), 8.4 (D), and 6.7 (E, F).

S3. Guinier plot

Figure S9 shows a Guinier plot of the scattering data at low q in Figure 7 in the Article. From the slope of the linear function fitted to the data the radius of gyration is calculated as $R_g = \sqrt{3 \cdot 1361.4}$ Å ≈ 63.9 Å

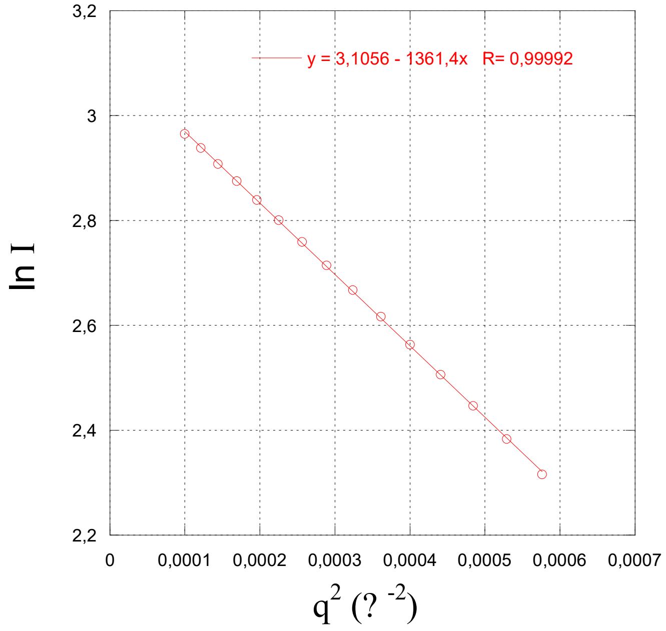

Figure S9. Logarithm of the scattering intensity in the low q-range (ln I) plotted vs. the square of the scattering vector (q^2) (Guinier plot). Data from synchrotron SAXS experiments on the shells formed in PSS macrogels after absorbing lysozyme from solution.

S4. Kinetic model

Consider the uptake of protein molecules by a spherical gel. The microscopy images indicate that a core-shell morphology is created and maintained during the course of binding. After an initial short period of volume decrease there is an extended period of much slower volume decrease. During the latter period the core-shell boundary continues to move toward the centre and the shell appears to become inhomogeneous. Interestingly, the rate of the moving boundary increases with decreasing (apparent) degree of crosslinking. It is proposed in the Article that the period of slow volume decrease begins at some degree of binding, denoted β', independent of the degree of crosslinking. If that is correct it follows from geometry that the thickness of the shell at β' will decrease with decreasing degree of crosslinking (largest intrinsic swelling) and therefore the rate of protein transport through the shell will increase. To test if this hypothesis can explain the experimental observations we derive here a kinetic model in which the change of the gel volume and growth of the shell is coupled to the rate of protein transport through the shell. For simplicity, we consider an idealized case where the inhomogeneous shell is replaced by two homogeneous layers with different compositions.

The model is derived based on the following assumptions. At each radial distance from the centre, freely mobile protein molecules are in equilibrium with protein molecules forming complexes with the network chains. The molecules in the latter state are immobilised and take

part of a dense "phase" permeable to the mobile protein molecules. During a first period, where the degree of protein binding increases from zero to β', the phase grows from the gel boundary and inward at constant network/protein charge ratio (f_s) and volume per network charge (v_s). In this way a sharp boundary between the "shell" and the protein-free "core" is maintained. The boundary moves toward the centre with a rate controlled by the rate of mass transport of mobile protein molecules from the liquid solution to core boundary. During this process the volume of the gel is given by the volume of a protein-free core and the volume of the shell as described by eq. (6) in the Article. When the degree of binding reaches β' the volume change of the gel stops. For $\beta > \beta$' the shell is unchanged but a 'core diffusion layer' (*cdl*) develops with a sharp diffusion front migrating toward the centre of the gel. Like in the shell, a fraction of the protein molecules are immobilised by their interaction with the network, and *cdl* grows inward at constant network/protein charge ratio (f_{cdl}) and volume per network charge ($v_{cdl} = v_0$). The rate of migration of the diffusion front is limited only by the rate of protein transport through the shell and the *cdl*.

The diffusive transport through a spherical surface is given by

$$\frac{dn}{dt} = -4\pi r^2 D \frac{dC}{dr} \tag{S4}$$

For steady state transport through a spherical layer one obtains after integration

$$\left(\frac{dn}{dt}\right)_{ss} = \frac{4\pi D r_1 r_2 (C_2 - C_1)}{r_1 - r_2} \tag{S5}$$

Consider a core/shell gel of radius r_2 and core radius r_1. Define the rate of incorporating lysozyme molecules into the shell as the rate of lysozyme molecules reaching r_1, thereby letting the core act as a sink ($C_1^{core} = C^{core} = 0$). By introducing the scaled radii defined in the Article we can then write:

$$\frac{d\beta_{shell}}{dt} = -\frac{Z}{n_p}\left(\frac{dn}{dt}\right)_{ss} = \frac{3ZP_s\bar{r}_1\bar{r}_2C_2}{C_{p,0}R_0^2(\bar{r}_2 - \bar{r}_1)} \tag{S6}$$

C_2 is the concentration of lysozyme in the liquid in contact with the gel, $C_{p,0}$ is the concentration of polymer network charges in the gel prior to binding, and R_0 is the initial gel radius. P_s is the permeability of the shell to lysozyme:

$$P_s = D_s K_s \tag{S7}$$

$$K_s = \frac{C_2^s}{C_2} \tag{S8}$$

with D_s being the diffusion coefficient in the shell, C_2^s the concentration of mobile lysozyme in the shell at r_2, and K_s a partition coefficient. In eq. (S6), $\bar{r}_1$ and $\bar{r}_2$ are considered to be functions of β given by eqs. (9) - (11) in the Article. Furthermore, we apply quasi-steady state conditions, meaning that steady state concentration profile is quickly reestablished for each shell thickness, i.e., for each pair $\{\bar{r}_1; \bar{r}_2\}$. It is straight forward to extend the model to include a second layer to describe the transport of lysozyme simultaneous through the (outer) shell and *cdl*. The rate of increasing the degree of binding to the gel becomes:

$$\frac{d\beta}{dt} = \frac{3ZP_1P_2\bar{r}_0\bar{r}_1\bar{r}_2C_2}{C_{p,0}R_0^2\{\bar{r}_2P_2(\bar{r}_1 - \bar{r}_0) + \bar{r}_0P_1(\bar{r}_2 - \bar{r}_1)\}} \tag{S9}$$

where r_0 is the position of the boundary between the core and *cdl*. Again, we have assumed that the concentration of lysozyme in the core is zero ($C_0^{core} = C^{core} = 0$), and introduced a permeability P_{cdl}:

$$P_{cdl} = D_{cdl} K_{cdl} \tag{S10}$$

$$K_{cdl} = \frac{C_1^{cdl}}{C_2} \tag{S11}$$

with D_{cdl} being the diffusion coefficient in *cdl*, C_1^{cdl} the concentration of mobile lysozyme in *cdl* at r_1, and K_{cdl} a partition coefficient.

By using eqs. (9) – (11) in the Article to substitute for $\bar{r}_0$, $\bar{r}_1$ and $\bar{r}_2$ in eq. (S9), the expression can be integrated to give relationships between time and β. The formation of the shell ends abruptly at a given β', after which no further deswelling and growth takes place. Thus, integration is made in two steps according to:

$$\beta \leq \beta': \quad \begin{cases} \bar{r}_0 = \bar{r}_1 \\ \beta = \beta_s \end{cases}$$

$$\beta > \beta': \quad \begin{cases} \bar{r}_1 = \bar{r}_1(\beta') \\ \bar{r}_2 = \bar{r}_2(\beta') \\ \beta_s = \beta' \end{cases}$$

For the first step ($\beta \leq \beta'$) the result is:

$$t = \frac{C_{p,0} R_0^2}{2Z f_s P_s C_2} \left\{ 1 - (1 - f_s \beta)^{\frac{2}{3}} - \frac{1 - (\bar{v}_s + (1 - \bar{v}_s)(1 - f_s \beta))^{2/3}}{1 - \bar{v}_s} \right\} = \frac{C_{p,0} R_0^2}{2Z f_s P_s C_2} \left\{ 1 - \bar{r}_1^2 - \frac{1 - \bar{r}_2^2}{1 - \bar{v}_s} \right\}. \tag{S12}$$

For the second step ($\beta > \beta'$) the result is:

$$t - t' = \frac{C_{p,0} R_0^2}{2Z f_{cdl} P_{cdl} C_2} \left\{ \bar{r}_1^2 - \left(\bar{r}_1^3 - f_{cdl}(\beta - \beta_s) \right)^{\frac{2}{3}} + \frac{2 f_{cdl}}{3} \left(\left(\frac{1}{\bar{r}_1} - \frac{1}{\bar{r}_2} \right) \frac{P_{cdl}}{P_s} - \frac{1}{\bar{r}_1} \right) (\beta - \beta_s) \right\} \tag{S13}$$

where t' is the time when $\beta = \beta'$.

References

1. Treloar, L. R. G. The Elasticity and Related Properties of Rubbers. *Rep. Prog. Phys.* **1973**, *36*, 755-826
